# Modeling glioblastoma

**DOI:** 10.7554/eLife.100824

**Published:** 2024-07-25

**Authors:** Ian Lorimer

**Affiliations:** 1 Ottawa Hospital Research Institute Ottawa Canada; 2 Departments of Medicine and Biochemistry, Microbiology and Immunology, University of Ottawa Ottawa Canada

**Keywords:** glioblastoma, cancer variant modeling, tumor microenvironment, inflammation, cancer, Zebrafish

## Abstract

Establishing a zebrafish model of a deadly type of brain tumor highlights the role of the immune system in the early stages of the disease.

**Related research article** Weiss A, D’Amata C, Pearson BJ, Hayes MN. 2024. A syngeneic spontaneous zebrafish model of *tp53*-deficient, EGFR^vIII^, and PI3KCA^H1047R^-driven glioblastoma reveals inhibitory roles for inflammation during tumor initiation and relapse in vivo. *eLife* **13**:RP93077. doi: 10.7554/eLife.93077.

Glioblastoma is the most common type of brain cancer in adults. Patients initially have relatively non-specific symptoms such as headaches, nausea and cognitive changes. Their diagnostic journey usually involves undergoing magnetic resonance imaging (MRI) of the brain, as this approach is highly effective at distinguishing glioblastoma from other conditions with similar symptoms. When the scan shows that glioblastoma is likely, patients typically undergo surgery; a definitive diagnosis comes from the examination of a surgical sample by a pathologist.

This dry clinical description glosses over the human and emotional toll of the disease. Patients quickly learn that while surgery can be helpful, it is never curative, and that radiation and chemotherapy have limited benefits and may also have serious side effects. Around half of glioblastoma patients die within 15 months of their diagnosis, and only about 5% are still alive after five years (Stupp et al., 2005). This cancer is a brutal disease that seems to come out of nowhere.

Glioblastoma is currently being intensively studied worldwide. Advanced genetic analyses have allowed researchers to better identify the mutations that contribute to the emergence of this cancer, as well as the different types of cells forming the tumor and the various ‘states’ they can adopt (Abdelfattah et al., 2022; Brennan et al., 2013; Neftel et al., 2019). However, a clear understanding of the very early stages of the disease has remained elusive. Now, in eLife, Alex Weiss, Cassandra D'Amata, Bret Pearson and Madeline Hayes report having established a zebrafish model of glioblastoma that provides insight into this critical period (Weiss et al., 2024).

Zebrafish are small animals that reproduce fast and are easy to raise in the laboratory. They are also naturally transparent early in development, allowing researchers to use microscopy techniques to observe cellular interactions in real time. Their genes are well-studied and can therefore be matched to their human counterparts based on sequence similarities, meaning findings made in this animal model could be translated into discoveries relevant to patients.

The team (who are based at the Hospital for Sick Children in Toronto, the University of Toronto and Oregon Health & Science University) worked with a line of zebrafish lacking a protein called TP53 that normally suppresses tumor development but is commonly inactivated in glioblastomas. The animals were genetically engineered so that mutated genes known to drive the emergence of glioblastoma were expressed in their neural progenitors, the cell type from which this cancer usually originates. This included two oncogenes, EGFR^vIII^ and PIK3CA^H1047R^, which prompt ‘rogue’ cells to multiply and invade healthy tissues. As a result, the fish reliably developed tumors similar to those found in glioblastoma patients. Notably, the experiments revealed a characteristic pattern of gene expression associated with inflammation, which has also been observed in human glioblastoma (Richards et al., 2021).

This result prompted Weiss et al. to investigate which immune cells were present in zebrafish tumors, with a focus on microglia, the resident macrophages of the brain. These cells are very abundant in human glioblastoma samples taken from large, established tumors, where they have an immunosuppressive role (Lee et al., 2021). In established zebrafish tumors, infiltrating microglia were readily detected. The cells adopted a rounded, ‘activated’ morphology and were observed to be engulfing and digesting glioblastoma cells, as they do in the human version of the disease (Penfield, 1925). Excitingly, Weiss et al. were able to show that microglia were present and active not only when a zebrafish tumor was well-established but also when it had just started to grow.

The team then aimed to assess the role of microglia in early glioblastoma using two different approaches. In the first set of experiments, precisely deleting one of two genes involved in the immune response – one required for macrophage and microglia development, the other for mediating inflammatory responses – resulted in tumors being more likely to form. Next, Weiss et al. introduced cells from zebrafish glioblastomas into healthy zebrafish embryos. When the transplanted embryos were administered clodronate, a drug that destroys microglia, their risk of growing tumors increased. Taken together, these findings suggest that these immune cells help suppress the development of the cancer early on (Figure 1B).

**Figure 1.**
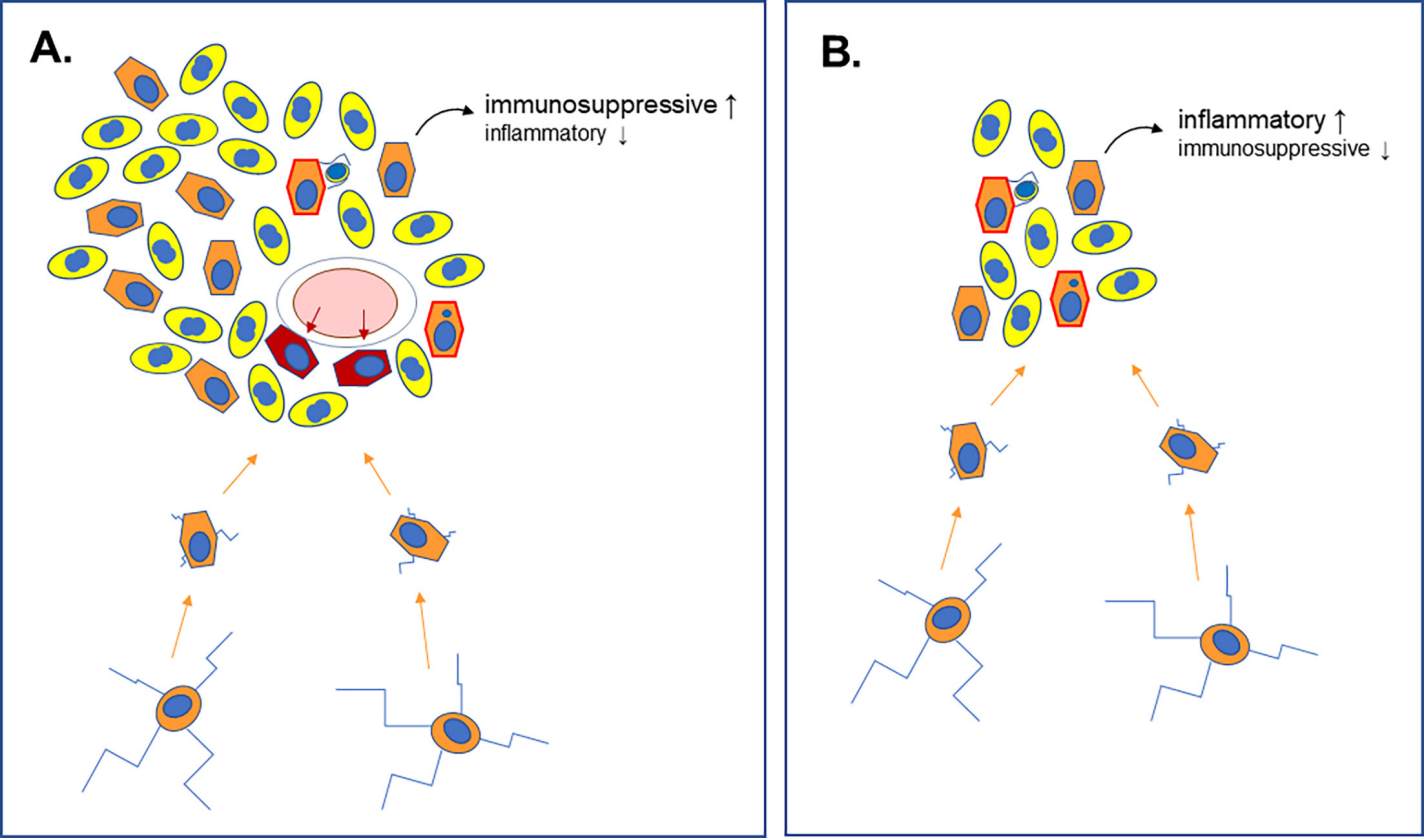
Different roles for microglia in late-stage and early-stage glioblastoma. (**A**) Analyses of late-stage glioblastoma tumors show that, along with tumor cells (yellow, with nuclei shown in blue), large numbers of microglia (orange, with nuclei shown in blue) are also present. Microglia in the normal brain have multiple long extensions (bottom cells with blue extensions). Upon recruitment into tumors (orange arrows), these are lost, and the cells adopt a rounder morphology. Late-stage glioblastoma tumors also develop new blood vessels (pink ellipse with white rim) from which they recruit macrophages (dark red). Microglia and macrophages engage in phagocytosis within the tumor (orange cells with red border), engulfing and digesting rogue cells. Multiple studies have shown that microglia are immunosuppressive and tumor-promoting in this context. (**B**) Weiss et al. used their zebrafish model to show that microglia are also recruited in the early stages of the disease, during which the cells also engage in phagocytosis. During this period, however, these cells do not help to suppress the immune system; instead, they increase the inflammatory response, and their depletion enhances glioblastoma development.

Current cancer therapies that rely on prompting the immune system to attack tumors are largely ineffective for glioblastoma (Reardon et al., 2020). This study points to a different role for microglia in the early stages of the cancer, when they actively repress tumor formation. Further studies are needed to uncover how these immune cells shift from suppressing to promoting the growth of this cancer as it develops. This knowledge may provide new avenues to develop effective glioblastoma immunotherapy and offer new hope to patients and their families.
